# Attachment and Adulthood in a Sample of Southeastern Mexico

**DOI:** 10.3390/bs9120134

**Published:** 2019-12-03

**Authors:** Gabriela Isabel Pérez-Aranda, Vianey Peralta-López, Sinuhé Estrada-Carmona, Liliana García Reyes, Miguel Angel Tuz-Sierra

**Affiliations:** 1 Project of Development to the Gender Perspective, Autonomous University of Campeche, San Francisco de Campeche 24039, Mexico; gaiperez@hotmail.com (G.I.P.-A.); saori_9414@hotmail.com (V.P.-L.); 2 Laboratory of Care and Clinical Research, Faculty of Humanities, Autonomous University of Campeche, San Francisco de Campeche 24039, Mexico; ligarcia@uacam.mx (L.G.R.); miantusi@hotmail.com (M.A.T.-S.)

**Keywords:** couple, attachment, early adulthood, middle adulthood

## Abstract

The main purpose of this research is to analyze the attachment styles of men and women from 20 to 40 years old (early adulthood) and from 40 to 65 years old (middle adulthood) who are in a romantic relationship and live in the City of San Francisco Campeche. A sample of 50 men and 50 women in early adulthood and 50 men and 50 women in middle adulthood was selected, and the questionnaire “Styles of Attachment” was applied. For the data analysis, we used the SPSS version 23 program along with student’s “t” and *X*^2^ (Chi^2^) tests. The results show no significant differences in attachment styles between male and female relationships. However, with respect to the stages analyzed, significant differences are found in attachment styles. In addition, there are significant differences with respect to the type of relationship: single people have secure attachment while married people display anxious attachment. We conclude that in early adulthood, secure attachment predominates, while in middle adulthood anxious attachment predominates.

## 1. Introduction

### Attachment in Couples

Once they are born, human beings come into contact with the outside world and have the need to establish a relationship of attachment with those who are close to them. Parents are the first to be present in a baby’s environment and are therefore the ones who have a responsibility to guarantee and meet his or her various needs, such as food, safety, affection, and love, in order to promote development in terms of psychological, physical, and social well-being.

Similarly, this attachment is the affective bond that is established between the newborn and his or her primary caregiver, and thanks to it, the child will be provided with the indispensable necessities for his or her development. Furthermore, this attachment has life-long effects: the attachment established at the beginning of life is so important that it can remain and will largely be the basis for affective relationships throughout life [1,2].

On the other hand, the concept of attachment refers to the willingness of a child or an older person to seek proximity and contact with an individual, especially under circumstances perceived as adverse. This arrangement changes slowly over time and is unaffected by situations of the moment [3]. The term attachment is used to refer to a property of human beings that exists from birth to death. The attachment system is innate and vital for survival, and lasts throughout development [4]. Although a variety of definitions of the term attachment have been suggested, this document will use the definition suggested by Bowlby [4], the main exponent of attachment theory, who understands this phenomenon as a process where a special bond is established and maintained with the individuals or people who have the ability to help one face one’s environment. This author proposes three types of attachment: secure, anxious, and avoidant, which also have great relevance in matters of couple relationships (the influence of these mediates and adjusts the degree of attachment intimacy). For Bowlby, this style of attachment is mainly characterized by conflicts generated by emotional closeness when in a couple [4].

The authors of [5] mention that attachment characteristics are associated with stages of human development; in this way, they state that during the first years of life, the intensity of a child´s attachment is related to a child’s interaction with caregivers and his or her environment. During the rest of childhood, this is specifically the case with caregivers, while during adolescence and adulthood, the main attachment figures are peers.

It has been reported that people with a secure attachment style will feel comfortable and confident being intimate, they will not need to be alert in their relationships, and they will simply be able to enjoy life and feel safe. Anxious people feel vulnerable to abandonment and are alert of any possible threat, while avoidants will try to avoid spaces of intimacy through strategies, separating erotic desire from its emotional implications [5]. Likewise, secure attachment is described as a type of satisfactory bond in a child, distinguished by emotional closeness to his or her caregiver in the immediate context, in which the child tolerates both the proximity and distance of the significant figure, a characteristic that allows him or her to live with the security that the caregiver will protect him or her against the adversities of the environment, which facilitates a rewarding context for psychic development [6].

For his part, Bowlby [6] states that, in anxious attachment, the infant is unsure if his parent will be sensitive and will help him when he needs it. It is characterized by experiencing anxiety about abandonment. This attachment style has a negative impact on relationships, which includes the continuous perception of the possibility of rupture or conflict, commonly accompanied by behaviors of protest, anger, and a lot of anguish regarding the separation of the object of love [7].

The authors of [8] maintain that adults who have anxious attachment do not have expectations of trust in an interpersonal context due to the emotional instability generated by contact with others; in this sense, although they possess an inherent desire to achieve some proximity with others, at the same time they feel too insecure to approach others, unconsciously seeking self-sabotage in the interpersonal plane, due to the poor interactions they have had with their attachment figure in childhood. On the other hand, the authors of [9] mention that avoidant attachment shares characteristics with positive attachment where there is no dependence; however, people with the former attachment style go on the defensive, they have expectations that others are not reliable, and they find intimate relationships threatening, and therefore they avoid and deny the need for intimacy.

It has been found that attachment styles remain stable over time; that is, they manifest within adult relationships as a style of love expressed by each member, a product of the experiences that a subject has had in childhood that have persisted throughout their life [10].

During childhood, the main attachment figures for a child are usually his or her parents; however, from adolescence onwards the attachment is directed towards other figures, thus, authors such as [11] state that relationships between couples share key aspects that allow them to be understood as relationships of attachment. In the partner relationship, the individual will develop a system of behaviors depending on the style of attachment he or she has learned. In this way, the paternal-filial bond and the couple relationship share key aspects and both can be considered as attachment relationships [12].

It has been observed that in adulthood, the process of searching for new attachment figures is motivated by a maturation at the social and sexual levels, which makes a partner relationship a favorable scenario for the development of attachment between people [13].

It has been reported that the adult attachment system works by seeking proximity at the beginning of a relationship and as internal working models in the selection of a partner, so dysfunctional attachment generates stress and anxiety [14].

On the other hand, a couple is a bond of affection, respect, companionship and love between two people; however, this bond may not be the healthiest for many people, as it will depend on the attachment styles present in their relationship as a couple.

For its part, [15] refers that the couple is a social organization that differs from the family in a relatively recent historical epoch; it fulfils a series of functions in the social fabric and therefore has rules and role prescriptions in relation to this social and historical background, i.e., the environment.

The couple relationship involves exploring the emotional map of the other person, capturing its limits and also detecting the borders and barriers it raises, while having the sensitivity activated to realize to what areas its “I” lets us access without discomfort and when it feels invaded or threatened. This empathic learning will be one of the basic aspects of living together in harmony. The authors of [16] define it as a joint space, which consists in forming an affective team that favors the personal growth of each of the members of the couple, through the deployment and updating of their potential for improvement and their learning capacity.

For [17], a starting point to understand the couple relationship is to consider that for human beings, more than for any other species, the needs of affection, attachment, care, affection, interdependence, companionship and love are genetically basic needs and determinants for the survival of the species.

During early adulthood (20 to 40 years), people may be in intimate relationships but they do not tend to be very durable. During middle adulthood (40 to 65 years), couple relationships are often in conflict due to the dual responsibility of caring for children and parents, while during the passage of the years the children marry and leave home, leaving the nest empty [18].

Studies indicate that people with secure attachments show the highest levels of satisfaction and involvement, while among insecure subjects there are the highest levels of dissatisfaction in partner relationships [19]. In themselves, insecure attachment styles tend to generate poor levels of affective communication, anxious ones tend to become defensively aggressive towards conflict resolution, while avoidant attachment styles simply avoid resolving them [19].

Another implication of attachment styles is their effects on the attitudes, emotions, and behavioral strategies in the relationship [20]; for example, individuals with anxious attachment tend to perceive more conflict within the relationship, therefore generating more stress, which ends up affecting the quality of the relationship because it damages the closeness and satisfaction in the couple.

In view of this, the present research consists of analyzing the differences in the attachment styles of men and women in a couple relationship, considering those who are in the early and middle adulthood stages, starting from the research question: What differences exist between early and middle adult men and women in relation to attachment styles? The initial hypothesis raises the existence of significant differences in the types of attachment between men and women.

## 2. Materials and Methods

This research has a non-experimental, cross-sectional, quantitative approach with a correlational scope. The objective was to analyze couple attachment styles in men and women in two stages of adulthood: emerging and medium.

### 2.1. Participants

The sample was located in the City of San Francisco de Campeche, specifically in the areas of Samulá, Sascalum, Centro, San Rafael, and Avenida López Mateos.

Two hundred questionnaires were applied to a non-probabilistic convenience sample, distributed the following way:

G1: 50 men in early adulthood, Men Early Adulthood (MEA) (between 20 and 40 years of age)

G2: 50 women in early adulthood, Women Early Adulthood (WEA) (between 20 and 40 years of age)

G3:50 men in middle adulthood, Men Medium Adulthood (MMA) (between 40 and 65 years of age)

G4: 50 women in middle adulthood, Women Medium Adulthood (WMA) (between 40 and 65 years of age)

### 2.2. Instruments

For the purposes of this research, the following instruments were administered:

(a) Personal data sheet, which includes: sex, age, schooling, type of relationship (single or married), marriage number, number of children along with their ages, and relationship length.
(b) Attachment style scale [21].

This scale evaluates attachment styles for couples: secure, anxious and avoidant attachment. It consists of 21 items, for which we obtained a reliability rating of 0.614 (Cronbach´s Alpha).

The first factor consisting of secure attachment has six reagents with a degree of reliability (Cronbach’s Alpha) of 0.837; anxious attachment has eight reagents and presents a degree of reliability (Cronbach’s Alpha) of 0.689; and finally avoidant attachment has seven reagents with a degree of reliability (Cronbach’s Alpha) of 0.687.

### 2.3. Data Procedure and Analysis

A convenience sample of 200 people was selected: 50 women in early adulthood, 50 women in middle adulthood, 50 men in early adulthood and 50 men in middle adulthood. The participants signed a letter of informed consent and confidentiality of personal data. All subjects gave their informed consent for inclusion before they participated in the study. The study was conducted in accordance with the Declaration of Helsinki, and the protocol was approved by the Ethics Committee of the humanities faculty and the postgraduate and research department of the Autonomous University of Campeche with the number project code 077/UAC/2016. All procedures followed were in accordance with the ethical standards of the responsible committee on human experimentation of the Autonomous University of Campeche, México; the national code of ethics for psychological research, the national and local law of health and with the Helsinki Declaration of 1975, as revised in 2000. Informed consent was obtained from all persons for being included in the study. Additional informed consent was obtained from all individuals for whom identifying information is included in this article. The Attachment Styles questionnaire [21] was used to measure attachment styles. The sample was located in the colonies mentioned above of the City of San Francisco de Campeche. After application, the data were captured in SPSS statistical program version 25. Also, data transformation was performed to find the means of attachment styles (secure attachment, anxious attachment and avoidant attachment). The results were analyzed using the SPSS statistical program. The following statistical tests were used: Student’s “t” test, ANOVA, post-hoc and Pearson Chi-square.

## 3. Results

The results obtained after the analysis of the data are shown below.

In Table 1, it is evident that there were no significant differences between the attachment styles of men and women (bilateral significance >0.05, data obtained through Student’s “t” test); there was also no dependence between attachment styles and genders, a figure obtained by the Chi-square statistical test. In women, there was a prevalence of anxious attachment and in men, of secure attachment.

As shown in Table 2, the results of the “t” test on attachment styles and adulthood stages, early and middle, show significant differences in the couple attachment styles of groups of men and women who are in the early adulthood stage and those in middle adulthood (bilateral significance <0.05). The secure attachment style predominates in people in early adulthood, and the anxious attachment style predominates in middle adulthood. As for the avoidant attachment style, no significant differences were found between the adulthood stages.

Table 3 shows the relationship that existed between marital status and the anxious attachment style, and the relationship between marital status and the secure attachment style (bilateral significance <0.05).

An analysis of Tukey’s Honestly Significant Difference Post Hoc Test variance was performed to understand the anxious style of attachment in the distinct types of relationships, which in this case were courtship, first marriage and more than one marriage. We obtained significant relationships (significant at 0.05) regarding the anxious attachment style and relationship types. During courtship, people have a prevalence of the secure attachment style, while in first marriages, there is a tendency towards the anxious attachment style (Table 4).

After analyzing attachment styles and relationship types, the Chi-square test found that the anxious attachment style depends on the type of relationship, i.e., courtship, first marriage, or more than one marriage (Table 5).

Table 6 explains the correlation between the variables of age, number of marriages, years of relationship, number of children and average age of children with secure, anxious and avoidant attachment styles. We found that the secure attachment style is positively correlated with marriage number, with a magnitude of −0.140* (0.048 Sig.), which is low. In the case of the anxious attachment style, correlations were obtained for age with a magnitude of 0.171* (0.015 Sig.), the years of relationship with a magnitude of 0.169* (0.017 Sig.), and the average age of children with a magnitude of 0.184** (0.009 Sig.). These variables (age, years of relationship and average age of children) had a magnitude equal to indifferent in relation to the anxious attachment style. In the case of avoidant attachment, no significant correlations were found.

## 4. Discussion

For the human being, attachment in pair relationships is of vital importance due to the importance of meeting the needs of affection, love, security, sexual satisfaction and companionship. Attachment styles in pair relationships are of great relevance, since they mediatize the degree of well-being in couples. The objective of this research was to analyze the attachment styles of men and women at two different stages of adulthood: early and middle.

Regarding the styles of couple attachment in men and women, our findings are consistent with the previous studies [22,23] and [24], who found no significant differences in the attachment styles of men and women.

However, various research works have noted statistically significant differences in the attachment styles of men and women, for example, [25] identified differences in the avoidant attachment style between men and women, with women being the most avoidant.

Others [26] have found that women are the ones who maintain a greater degree of avoidant style and a lesser degree of secure attachment compared to men. Similarly, previous studies have indicated that there are differences between men and women in the association between dimensions of attachment and satisfaction with the couple relationship [27]. These gender differences have been associated with affective history: [19,28] found significant differences in the attachment of men and women, with men having a predominance of secure attachment, while in women anxious attachment, associated with dependence, predominates.

In this research, although without significant differences, women presented scores where anxious attachment was prevalent and men presented scores in which attachment was secure. In general, Mexican women are characterized by anxious/concerned style [29].

In a research study on jealousy, addictive love and attachment styles, [30] found that “concerned” attachment styles are associated with addictive love in women. Similarly, another author in [31] relates concerned couple attachment to women. Also, in [29] it was found that women who are victims of marital violence exhibit a concerned style of attachment, characteristics that negatively influence the confrontation of abuse and, consequently, the maintenance of the abuse.

Others [24] analyzed the interaction between attachment profiles, the degree of intrapersonal conflict with sexual desire and the different types or ways of exercising care for the other, all within the scope of the couple system, and described a relationship between anxious attachment and an over-activation of care, which is associated with the difficulty of emotional regulation for anxious people.

Self-image is related to the degree to which one experiences anxiety about being rejected or abandoned, so that people who have a positive vision of themselves tend to experience low anxiety about this possibility, given that they are considered worthy to be loved and cared for [32].

Women, unlike men, are more satisfied when the two have a secure attachment than when only she has secure attachment and he has anxious; concerned men are not able to value the support of a secure person whereas concerned women are. Therefore, the satisfaction of an anxious partner will depend on the attachment style and the sex of their partner [33].

These research studies explain the predominance of the anxious attachment style in women and reveal the relevance of how attachment styles can impact women’s mental, physical and sexual health, as well as the way in which this style of attachment in women could mediate relationships and their satisfaction and well-being.

Regarding the two-stage attachment styles of adulthood, in the sample of this research, there were significant differences in the attachment styles of the group of people in early adulthood and that of people in middle adulthood. 

Secure attachment predominates in early adulthood and anxious attachment in middle adulthood; this is consistent with [22], who found that the anxious attachment style predominates in early adulthood.

Similarly, [29] offers an explanation and mentions that over time the frequency of the avoidant-independent style increases, while that of the secure-independent decreases, and notes that these results reflect the important behavioral effects that socialization towards independence, dynamism and autonomy exercises over Mexican men.

Likewise, [34] explains that the experiences of young adults in couple relationships are predominantly satisfactory, being greater in the case of relationships of greater stability and commitment, regardless of the time they have been involved in them, which is expected in this evolutionary period. This satisfaction could be associated with the fact that loving relationships in emerging adulthood present multiple alternatives and denominations, depending on differential levels of emotional involvement, exclusivity, sexual intimacy, permanence in relationship and its formalization [35].

The anxious attachment style in middle adulthood could be related to the greater responsibilities as a couple in middle adulthood with the arrival of children, and the couple living through these adjustments that most of the time limit their interaction [36].

These findings are contradictory to what is found by [37], who claim that relationships of greater commitment and stability tend to occur more frequently as young people approach the end of emerging adulthood. Similarly, anxious attachment is related to age (which is consistent with attachment styles in the early and middle adulthood stages), number of children, and age of children.

These results match what was found in [38], which describes that marriages perceive a decrease in their marital satisfaction as the number of children and the years of relationship increase. This study also describes that, considering the great overhead of roles and multifunctionality experienced by today’s couples, especially women, it might be expected that couples who do not have children would be less likely to have stress, and therefore more satisfaction in the relationship. On the contrary, [24] found no differences in satisfaction according to the presence of children, age, schooling, religious affiliation, or length of the marital relationship depending on the styles of attachment.

The secure attachment style was found to be related to the number of marriages: with a greater number of marriages, the more prevalence of secure attachment.

The results obtained with respect to the attachment styles of men and women, and the type of relationship (courtship, first marriage and second marriage), indicate that people who are in a courtship relationship possess a secure style of attachment and people who are in a first marriage have an anxious attachment style, while there were no significant differences for the attachment styles of people in a second marriage. Regarding marital status: married and single, married people presented an anxious style of attachment while singles a secure style of attachment.

These results coincide with what was identified in [11]: the dimension of intimacy was higher than the anxiety dimension in the married women’s group, meaning that they have a secure style of attachment; similar data were found in divorced women, who had a higher average in the anxiety dimension compared to the group of married women.

Finally, it is important to conclude by describing the dependence that existed in this research between the anxious attachment style and the types of relationship, where, as mentioned above, the anxious attachment style was related to marriage.

The understanding of marital satisfaction may be related not only to the characteristics of the individual, but also to those of the couple and what emerges in that space link [24]. People with secure attachment styles control their negative feelings in a relatively constructive way, recognizing their anxiety and seeking support or comfort in the couple.

## 5. Conclusions

For the human being, it is vitally important to establish couple bonds since we need to satisfy needs of affection, love, security, sexual satisfaction and company. The attachment styles in couple relationships are of great relevance in couple issues, since they mediate the degree of well-being within such relationships. The results of the present investigation allow us to affirm that there are no differences in the attachment styles of men and women. However, there were significant differences in attachment styles in relation to the stages of development. Secure attachment was found in early adulthood and anxious attachment in middle adulthood. On the other hand, people in a courtship have a tendency towards secure attachment, and those who are married towards anxious attachment.

It is important to mention that the results of this research may be consulted as background for future research related to the variable studied, i.e., attachment styles.

## 6. Limitations and Future Research Perspectives

For subsequent studies, it is recommended to work with other stages of development, such as adolescence and late adulthood, and expand the sample size so that a better understanding of the results and generalization of the data can be achieved.

## Figures and Tables

**Table 1:** Dependency analysis and comparison of the means of attachment styles according to sex.

			*t*-Test				
Attachment	Gender	Mean	*t*	Sig.	Count	X^2^	Sig.
Secure	Men	6.0317	1.440	0.151	92	2.190	0.335
	Women	5.7883			87		
Anxious	Men	3.7125	0.963	0.337	7		
	Women	3.5513			9		
Avoidant	Men	2.3071	0.102	0.919	1		
	Women	2.2914			4		

**Table 2:** Dependency analysis and comparison of the averages of attachment styles according to adulthood stage (early: EA, middle: MA).

			*t*-Test				
Attachment	Stage	Mean	*t*	Sig.	Count	X^2^	Sig.
Secure	EA	6.0833	2.063	0.040 *	94	4.503	0.105
	MA	5.7367			85		
Anxious	EA	3.4575	2.100	0.037 *	5		
	MA	3.8063			11		
Avoidant	EA	2.3957	1.255	0.211	1		
	MA	2.2029			4		

* (*p* < 0.05).

**Table 3:** Comparison of the averages of attachment styles according to marital status.

Type of Attachment	Marital Status	Mean	*t*	Sig.
Secure	Single	6.0892	2.113	0.036 *
	Married	5.7343		
Anxious	Single	3.4533	−2.130	0.034 *
	Married	3.8069		
Avoidant	Single	2.3954	1.238	0.217
	Married	2.2051		

* (*p* < 0.05).

**Table 4:** Tukey’s Honest Significant Difference post-hoc variance analysis of the anxious style according to relationship types.

Attachment Style	Type of Relationship	Type of Relationship	Mean	Typical Error	Sig. (Bilateral)
Anxious	Courtship	First marriage	3.9096	0.18689	0.043 *	
		More than one marriage	3.6143	0.23038	0.775	
	First marriage	More than one marriage	3.6143	0.24593	0.454	

* (*p* < 0.05).

**Table 5:** Dependency analysis through X^2^ between attachment styles and relationship type.

Attachment	Courtship	First Marriage	More than One Marriage	X^2^	Degrees of Freedom	Sig.
Secure	94	53	32			
Anxious	5	10	1	1	9.847	0.043 *
Avoidant	1	2	2			

* (*p* < 0.05).

**Table 6:** Pearson correlation analysis between sociodemographic variables and attachment styles.

Variables		Types of Attachment		
		Secure	Anxious	Avoidant
Age	Pearson Correlation Sig. (bilateral)		0.171 * 0.015	
Number of Marriages	Pearson Correlation Sig. (bilateral)	−0.140 * 0.048		
Years of Relationship	Pearson Correlation Sig. (bilateral)		0.169 * 0.017	
Number of Children	Pearson Correlation Sig. (bilateral)			
Average Age of Children	Pearson Correlation Sig. (bilateral)		0.184 ** 0.009	

* (*p* < 0.05), ** (*p* < 0.001)
